# Evaluation of ReachOut.com, an Unstructured Digital Youth Mental Health Intervention: Prospective Cohort Study

**DOI:** 10.2196/21280

**Published:** 2020-10-15

**Authors:** Bianca Lorraine Kahl, Hilary May Miller, Kathryn Cairns, Hayley Giniunas, Mariesa Nicholas

**Affiliations:** 1 ReachOut Australia Pyrmont Australia

**Keywords:** digital mental health, digital intervention, youth, internet-based intervention, depression, anxiety, stress, suicide

## Abstract

**Background:**

Young people experience a disproportionate burden associated with mental illness that Australia’s mental health care system is ill-equipped to handle. Despite improvements in the provision of mental health services, the rates of service utilization among young people remain suboptimal, and there are still considerable barriers to seeking help. Digital mental health services can overcome a number of barriers and connect young people requiring support; however, the evidence base of digital interventions is limited.

**Objective:**

The aim of this study is to examine the effectiveness of a brief, self-directed, unstructured digital intervention, ReachOut.com (hereafter ReachOut), in reducing depression, anxiety, stress, and risk of suicide.

**Methods:**

A cohort of 1982 ReachOut users participated in a 12-week longitudinal study, with a retention rate of 81.18% (1609/1982) across the duration of the study. Participants completed web-based surveys, with outcome measures of mental health status and suicide risk assessed at 3 time points across the study period.

**Results:**

The results demonstrated that over the 12-week study period, young people using ReachOut experienced modest yet significant reductions in symptoms of depression, anxiety, and stress. Significant, albeit modest, reductions in the proportion of participants at high risk of suicide were also observed.

**Conclusions:**

The findings of this research provide preliminary evidence of the promise of an unstructured digital mental health intervention, ReachOut, in alleviating symptoms of mental ill-health and promoting well-being in young people. These findings are particularly important given that digital services are not only acceptable and accessible but also have the potential to cater to the diverse mental health needs of young people at scale, in a way that other services cannot.

## Introduction

### Background

Young people experience a high burden of disease attributable to mental ill-health, with approximately one in four young people (aged 15-24 years) experiencing a mental health problem every year [1]. Unfortunately, the prevalence of psychological distress in young people in Australia appears to be increasing [2], and suicide remains the leading cause of death for Australian youth [3]. Many mental health problems have their onset in adolescence or early adulthood, and if left untreated, can result in more frequent and severe episodes of ill-health throughout the lifespan [4-7]. There is limited capacity within the current mental health service system to meet the demands associated with youth mental health problems in a timely manner, and many young people are going without vital support [8].

Although there have been steady improvements in the provision and uptake of treatment services among young people in the past decade, rates of service utilization among adolescents and young adults remain suboptimal; in the latest Australian Child and Adolescent Survey of Mental Health and Wellbeing, 21.4% of young people aged between 12 and 17 years had accessed a service for emotional or behavioral problems in the past 12 months [8,9]. Furthermore, despite increased provisions and extensive public destigmatization efforts, a significant proportion of young people are reluctant to access community services for their mental health problems [10,11]. Common barriers to help-seeking for young people include limited service availability (particularly in rural areas), accessibility issues (in terms of location and time), financial constraints, concerns about confidentiality, and fear of judgment or stigma [10,12,13]. A desire for autonomy in the help-seeking process and a preference for self-reliance are also salient barriers for young people [10,14,15]. In addition to these common barriers, there are a number of young people who report having unfavorable experiences with mental health professionals and are therefore hesitant to either return or seek further professional help [16]. In the light of these barriers, innovative approaches to reaching and engaging, or reengaging, young people with mental health support are required.

Digital mental health services have the potential to overcome common barriers to help-seeking experienced by young people [17]*.* Recent research indicates that young people perceive web-based mental health support to have many benefits over traditional face-to-face services, including greater accessibility, immediacy, interactivity, lower financial costs, and reduced embarrassment associated with the experience [18]. The use of internet among young people is prolific [19] and there is growing evidence that young people are turning to digital platforms to access mental health information and support [2]. Australian research indicates that approximately one in five 12 to 17-year-old adolescents, and one in three 18 to 25-year-old adults who have experienced a mental health problem have used internet search engines to access mental health information [20]. A national survey of young Australians conducted in 2018 [2] also found that young people were amenable to using the internet for mental health support. For example, 37% of those with psychological distress said that they would use the internet to source information about specific issues and 22% reported that they would use the internet to access information about available services [2]. Another recent study found that the majority of youth (72%) would access a web-based resource if they were experiencing a mental health problem, and a third (32%) would prefer a web-based resource over face-to-face therapy [18].

The flexible, freely accessible, and multicomponent nature of unstructured digital services may be appealing to young people experiencing issues more broadly; they may also be particularly attractive to young people with mental ill-health who are reluctant to engage with clinical services for several reasons. First, they can be accessed anonymously, whereas most structured programs require users to provide personal information, thereby circumventing concerns about confidentiality [2,10]. Second, they are self-directed and therefore responsive to young people’s preference for autonomy by providing a range of resources to select from [10]. Third, they are able to deliver a service experience that is tailored to the needs and preferences of each individual young person through personalization [21]. Finally, there are many evidence-based digital mental health services that can be accessed for free or at a low cost, with no waiting period [22,23]. Digital services might also act as an alternative or adjunct intervention when young people have had previous negative experiences with mental health professionals. In such cases, digital services may be well-positioned to provide interim support and rebuild service readiness in young people who are in need of more intensive intervention but are dubious about returning to professional services [24].

The growth in the accessibility and uptake of digital technologies offers a cost-efficient and highly scalable alternative to reach young people needing mental health support when compared with traditional models of clinical care [25]. Digital services have the potential to reach and deliver positive outcomes across the mental health spectrum. Moreover, they can be appropriate for young people who experience subthreshold mental health problems and who may be ineligible for other supports, or those who are reluctant to seek professional help owing to minimization and/or self-stigma. Currently, several digital support services exist to support young people in managing their mental health, including web-based counseling services (eg, eheadspace), structured web-based therapies (eg, Moodgym), psychoeducational websites, and web-based blogs and forums [26]. There is also increasing evidence of the effectiveness of web-based counseling, therapies, and mobile self-monitoring tools in improving mental health outcomes and help-seeking [27-33]. Web-based positive psychology interventions have also been linked to increased well-being and decreased stress in nonclinical youth populations [34]. Not only is there evidence of the effectiveness of digital services in the prevention and early intervention space but there is also evidence that mobile apps designed to prevent suicide can bring about reductions in psychological distress among vulnerable populations (eg, Ibobbly, [35]). Moreover, web-based interventions may also prevent relapse in mental illness and support a process of recovery [36,37]. Given the flexibility and versatility of digital media, digital services are well placed to support the broad spectrum of unique mental health issues that young people, both well and unwell, experience.

Although there is substantial evidence for structured and clinician-led digital interventions, limited research has been conducted to investigate the effectiveness of unstructured digital mental health interventions, and there is a lack of ecologically valid research for digital interventions. Although randomized control trials (RCTs) represent the gold standard in research and provide important insights for many treatment outcomes, a reliance on these methods means that much evaluative research of digital programs is conducted under highly controlled and prescriptive conditions that rarely mimic the experience of users in the real world [38]. To adequately evaluate the effectiveness of digital interventions, it is imperative to examine these digital services in the environments in which they are intended to be used [38]. This study aimed to bridge this gap by exploring the impact of one unstructured digital mental health intervention, ReachOut.com (hereafter ReachOut), on mental health outcomes of young people who used it over a 3-month period, in a naturalistic study.

### This Research

Preliminary findings from previous cross-sectional evaluations of ReachOut have suggested that the service has a positive impact on well-being [39,40]; however, further research is needed to investigate its effectiveness in improving the mental health and well-being outcomes of young people. This research aims to add to the extant evidence base for digital mental health interventions by critically evaluating the impact of ReachOut on symptoms of depression, anxiety and stress, and suicidal ideation in a large cohort of users.

## Methods

### Overview

A longitudinal study was conducted over a 12-week period to explore whether participating in a brief, unstructured digital intervention would be associated with changes in symptoms of depression, anxiety, stress, and suicide risk. Outcome measures of mental health status and suicide risk were assessed at 3 time points across the 12-week study period. We also briefly explored the user experience ratings of the service. This study was approved by the University of Melbourne Human Research Ethics Committee.

### Intervention

ReachOut is an unstructured, self-administered digital mental health service for young people aged 14 to 25 years. ReachOut resources are developed with the involvement of young people and are reviewed by a Clinical Advisory Group, composed of a diverse team of clinicians. ReachOut is accessed by more than 2 million people in Australia annually (based on visitation statistics from January 2020), which indicates that this unstructured digital service modality holds appeal for young people. ReachOut is a multi-layered service with components that vary in intensity. At the base level, it provides psychoeducational information, personal stories, quizzes, videos, and audio recordings. It also offers apps and tools, peer-support from both peers and trained moderators, pathways to clinical support, and customized recommendations to users through NextStep [41]. ReachOut is designed to support well-being affected by everyday stressors to more complex mental health issues, by providing evidence-informed self-help strategies, sharing stories of recovery, building knowledge of professional help, and increasing self-efficacy to seek help. We expected using ReachOut as part of this intervention to increase professional help-seeking behavior and reduce the impact of mental health symptoms.

### Materials

#### Mental Health Status

The mental health status of participants was assessed using the short form of the Depression, Anxiety, and Stress Scale (DASS-21 [42]). The DASS-21 comprises 21 items, which are considered over the past week, and make up three subscales that measure levels of depression (eg, “I felt that life was meaningless”), anxiety (eg, “I felt I was close to panic”), and stress (eg, “I found it difficult to relax”). Items were scored on a 4-point scale, where lower scores indicated lower severity (0=*Did not apply to me at all* to 3=*Applied to me very much, or most of the time*). Each subscale was scored and classified as indicating normal, mild, moderate, severe, or extremely severe levels of depression, anxiety, and stress. The measure demonstrated good reliability on the subscales of depression (α=.93), anxiety (α=.86), and stress (α=.87) at baseline.

#### Suicide Risk

Suicidal ideation was assessed using 8 critical items from the Reynolds’ Suicidal Ideation Questionnaire (SIQ) [43]. The 8 critical items scale included statements such as “I thought about how I would kill myself,” and were measured on a 7-point scale (0=*I never had this thought* to 6=*Almost every day*). These 8 items have demonstrated ecological validity, having been significantly correlated with clinical assessments of suicidality (*P*=.05, [44]). A score of 5 or 6 on more than 3 items of the 8 critical items is considered to indicate a higher risk of suicide in adolescents [43].

#### User Experience

Participant impressions of ReachOut were assessed using bespoke measures that align with the user experience goals for the service, which have been co-designed with young people. These items addressed the relevance, availability*,* and accessibility of *ReachOut*, whether ReachOut had helped participants understand their own experiences and whether it had provided them with helpful strategies and tools. These statements were ranked on a 5-point scale (1=*strongly agree* to 5=*strongly disagree*). Participants were also asked to rate their satisfaction with ReachOut on a 4-point scale (1=*poor* to 4=*excellent*).

### Recruitment

Participants were recruited through a pop-up notification on the ReachOut website. On clicking the notification, users were asked to complete an eligibility questionnaire. Participants were required to be residing in Australia, aged between 16 and 25 years, and to have used the ReachOut website previously, either to access information or support for themselves or for someone they knew. If eligible, participants were presented with an information statement for providing informed consent. Rolling recruitment occurred across two phases. Phase one recruitment commenced in November 2014 and ended in August 2015. Following this, phase two recruitment commenced in February 2016 and was completed in June 2016.

### Procedure

Participants completed 4 surveys across a 3-month period. A baseline survey (T1) captured demographic characteristics, mental health, and suicide risk. Further surveys were administered 1 week post baseline (T2), 5 weeks post baseline (T3), and 12 weeks post baseline (T4). User experience, including satisfaction with and impressions of the ReachOut website, were assessed at T2, using bespoke measures taken from past ReachOut user studies. Mental health and suicide risk were not assessed at T2. T3 and T4 repeated all measures from the baseline survey, excluding demographics. Each survey took 15-40 min to complete: Baseline (30-40 min), T2 (10-15 min), T3 and T4 (20-30 min).

### Statistical Analyses

Frequency and descriptive analyses were conducted to explore the characteristics of the participants. To explore whether participants experienced a reduction in depression, anxiety, and/or stress across the 12-week unstructured intervention study, a series of 3-way mixed analysis of variance (ANOVA) was conducted. These analyses were also used to determine whether the trends across time differed based on gender, sexual orientation, and age group. The assumption of sphericity was violated for all 3 analyses; therefore, the Greenhouse-Geisser correction was applied. The 3-way interactions were examined to explore whether reductions in depression, anxiety, and stress significantly differed between gender, sexual orientation, and age groups. We used Cochran’s Q analysis to explore whether there was a reduction in the proportion of ReachOut users at high risk of suicide across the 12-week study period. Finally, to explore changes in the proportion of people at risk of suicide by gender, sexual orientation, and age group, data files were split by group, and analyses were run separately as above.

## Results

### Participant Characteristics

A final sample of 1982 young people aged between 16 and 25 years (mean 19.40, SD 2.98) was recruited. The study retention rate was 81.18% (1609/1982), with 1609 participants completing the 3-month follow-up survey. Characteristics of all participants compared with those included in the analysis are shown in Table 1. No major differences were observed between those included and those not included in the analysis. However, those participants who self-selected for this study may not be representative of all people who use ReachOut more broadly. The sample was predominantly female, and many had previously sought help from a mental health professional. Of the participants who had sought help, only 54.53% (656/1203) said they found the help they received from the professional to be helpful. A relatively large number also had a history of mental health-related hospital admissions. See Table 1 for a full description of participant characteristics.

**Table 1:** Participant baseline characteristics.

Characteristics		Full sample (n=1982), n (%)	Analyzed sample (n=1609), n (%)
**Age (years)^a^**			
	16-18	915 (47.0)	759 (47.4)
	19-21	523 (26.9)	437 (27.3)
	22-25	509 (26.1)	406 (25.3)
**Gender identity**			
	Female	1657 (83.6)	1352 (84.0)
	Male	233 (11.8)	179 (11.1)
	Other (gender diverse)	92 (4.6)	78 (4.8)
**Sexual orientation^b^**			
	Heterosexual	1268 (65.0)	1050 (65.3)
	Lesbian/gay	103 (5.3)	90 (5.6)
	Bisexual	254 (13.0)	197 (12.3)
	Unsure/questioning	166 (8.5)	141 (8.8)
	Other/different	159 (8.2)	129 (9.0)
**Previous help-seeking**			
	Previously seen a mental health professional	1487 (75.0)	1203 (74.5)
	No	495 (25.0)	406 (25.2)
**Mental health hospitalizations**			
	No hospitalization	1667 (84.1)	1366 (84.9)
	Previous hospital admission for a mental health issue	289 (14.6)	223 (13.9)
	Don’t know	26 (1.3)	20 (1.2)
**User experience ratings of ReachOut (% agree or strongly agree)^c^**			
	ReachOut is relevant	1559 (82.3); N=1894	1327 (83.1); N=1597
	ReachOut is available and accessible	1646 (86.7); N=1899	1396 (87.5); N=1596
	ReachOut helps me to understand my own experiences	1361 (71.5); N=1903	1165 (72.9); N=1599
	ReachOut has given me a range of practical self-help strategies and tools	1303 (68.6); N=1899	1103 (69.2); N=1595
	Overall rating of ReachOut (% good or excellent)	1902 (99.0); N=1922	1588 (99.1); N=1602

^a^Not all participants reported age. Full sample (N=1947), analyzed sample (N=1602).

^a^Not all participants reported sexual orientation. Full sample (N=1950), analyzed sample (N=1607).

^c^Not all participants responded to the user experience *ratings.*

### Changes in Depression, Anxiety, and Stress Over Time

ANOVA revealed a significant main effect of time, showing that there was a significant decrease in overall DASS-21 scores from baseline to follow-up. There was also a significant main effect of DASS-21, highlighting differences in scores between depression, anxiety, and stress. However, these main effects were superseded by significant 2-way interactions between DASS-21 and time. Although scores on depression, anxiety, and stress decreased over time, depression showed the largest decrease from baseline to the 3-month follow-up, when compared with anxiety and stress. See Table 2 for means and standard deviations for interaction effects of DASS and time, gender, sexual orientation, and age group from the ANOVA models described below.

**Table 2:** Means and standard deviation for depression, anxiety, and stress across time, gender, age group, and sexual orientation.

Parameter		Depression, mean (SD)	Anxiety, mean (SD)	Stress, mean (SD)
**Time**				
	Baseline	22.55 (12.30)	16.53 (10.66)	21.96 (10.47)
	Follow-up	17.70 (12.74)	13.75 (10.44)	19.09 (11.00)
**Gender**				
	Male	20.07 (11.06)	13.47 (9.49)	18.81 (9.49)
	Female	19.46 (11.07)	15.09 (9.47)	20.43 (9.47)
	Other identity	22.52 (11.06)	16.86 (9.48)	22.53 (9.48)
**Sexual orientation**				
	Heterosexual	18.06 (10.85)	13.86 (9.38)	19.33 (9.41)
	Gay or lesbian	22.00 (10.85)	16.97 (9.37)	21.74 (9.40)
	Bisexual	22.48 (10.86)	16.37 (9.37)	22.24 (9.40)
	Unsure/questioning	23.04 (10.85)	17.39 (9.36)	21.90 (9.40)
	Different sexual identity	23.32 (10.86)	18.31 (9.36)	22.34 (9.40)
**Age (years)**				
	16-18	20.16 (11.07)	15.99 (9.46)	20.48 (9.49)
	19-21	18.91 (11.06)	14.41 (9.44)	20.16 (9.51)
	22-25	19.73 (11.05)	13.88 (9.45)	20.49 (9.49)

The 3-way interaction between gender, DASS-21, and time was not statistically significant (Table 3), nor was the 3-way interaction between sexual orientation, DASS-21, and time (Table 4). The 3-way interaction between age group, DASS-21, and time was also not statistically significant (Table 5). This demonstrates that the changes in depression, anxiety, and stress scores across time did not significantly differ with respect to gender, sexual orientation, or age group.

**Table 3:** Repeated measures effects of DASS across time, by gender.

Parameter	*F* test (*df*)	*P* value	η_p_^2^
DASS^a^	186.02 (1.78,2780.46)	<.001	0.106
Time	46.04 (1.93,3020.69)	<.001	0.029
Gender	3.21 (2.00,1565.00)	.04	0.004
DASS×Time	15.96 (3.75,5865.91)	<.001	0.010
DASS×Gender	5.53 (3.55,2780.46)	<.001	0.007
Time×Gender	0.28 (3.86,3020.69)	.89	0.000
DASS×Time×Gender	1.40 (7.50,5865.91)	.20	0.002

^a^DASS: Depression, Anxiety and Stress Scale-21

**Table 4:** Repeated measures effects of DASS across time, by sexual orientation.

Parameter	*F* test (*df*)	*P* value	η_p_^2^
DASS^a^	274.53 (1.77,2773.93)	<.001	0.150
Time	62.00 (1.93,3017.65)	<.001	0.038
Sexual Orientation	15.06 (4.00,1561.00)	<.001	0.037
DASS×Time	25.15 (3.75,5852.35)	<.001	0.016
DASS×Sexual Orientation	3.53 (7.11,2773.93)	.001	0.009
Time×Sexual Orientation	0.85 (7.73,3017.65)	.55	0.002
DASS×Time×Sexual Orientation	0.87 (15.00,5852.35)	.60	0.002

^a^DASS: Depression, Anxiety and Stress Scale-21.

**Table 5:** Repeated measures effects of DASS across time, by age group.

Parameter	*F* test (*df*)	*P* value	η_p_^2^
DASS^a^	539.53 (1.77,2752.98)	<.001	0.257
Time	155.33 (1.93,3010.53)	<.001	0.091
Age group	2.15 (2.00,1558.00)	.20	0.003
DASS×Time	38.34 (3.75,5843.55)	<.001	0.024
DASS×Age group	7.43 (3.53,2752.98)	<.001	0.009
Time×Age group	2.74 (3.87,3010.53)	.03	0.004
DASS×Time×Age group	1.08 (7.50,5843.55)	.37	0.001

^a^DASS: Depression, Anxiety and Stress Scale-21.

Although there were no statistically significant 3-way interactions, there were several statistically significant 2-way interactions. A statistically significant 2-way interaction was observed between the DASS-21 and gender. The differences between depression and anxiety *(F*_2,1565_=6.51, *P*=.002, η_p_^2^=0.008) and between depression and stress (*F*_2,1565_=7.94, *P*<.001, η^2^=0.010) differed based on gender. Participants who identified with the *other* gender identity consistently scored the highest on depression, anxiety, and stress. However, while female participants scored higher on anxiety and stress compared with male participants, male participants scored higher on depression when compared with female participants (Table 2 for means and standard deviations).

Furthermore, although there was a statistically significant 2-way interaction between DASS-21 and sexual orientation, upon further inspection of the effect, there did not appear to be a meaningful interaction and it did not demonstrate clear patterns between sexual orientation and the DASS-21. That is, heterosexual young people consistently scored lower for depression, anxiety, and stress, while lesbian or gay and bisexual young people consistently scored lower than those with the *other* sexual orientation. However, there were no other consistent trends between participants who were identified as gay or lesbian, bisexual, or unsure or questioning in depression, anxiety, or stress scores (Table 2 for means and standard deviations). This lack of interaction could have been caused by low participant numbers in the various groups.

Moreover, there was a statistically significant 2-way interaction between age group and time, from baseline to follow-up (*F*_2,1558_=3.06, *P*=.05, η^2^=0.004). That is, although all age groups showed a decrease in overall DASS-21 scores from baseline to the 3-month follow-up, this decrease was most pronounced for participants aged between 19 and 21 years (baseline mean 20.17, SD 9.94; follow-up mean 15.94, SD 10.44) when compared with those aged between 16 and 18 years (baseline mean 20.46, SD 9.92; follow up mean 17.47, SD 10.44) and participants aged between 22 and 25 years (baseline mean 20.01, SD 9.93; follow-up mean 16.77, SD 10.44).

There was also a statistically significant 2-way interaction between the DASS-21 and age groups. Contrasts revealed that there were differences in scores between depression and anxiety across age groups (*F*_2,1558_=5.70, *P*=.003, η_p_^2^=0.007) and between anxiety and stress across age groups (*F*_2,1558_=18.44, *P*<.001, η_p_^2^=0.023). Although the trends across age groups were similar for depression and stress, they differed for anxiety. Participants aged 16 to 18 years scored the highest for anxiety, followed by those aged 19 to 21 years, while those aged 22 to 25 years scored the lowest on anxiety (Table 2 for means and standard deviations).

### Changes in Suicidal Ideation Over Time

A total of 1577 participants completed the SIQ at baseline, 5 weeks postbaseline, and at the 3-month follow-up. There was a statistically significant difference between the proportions of participants at high risk of suicide across the different time points (χ^2^_2_=23.1, *P*<.001). About 12.30% (194/1577) were at high risk of suicide at baseline, 10.53% (166/1577) at 5 weeks postbaseline, and 8.50% (134/1577) at the 3-month follow-up. Pairwise comparisons were performed using Dunn’s procedures with a Bonferroni correction for multiple comparisons, presenting adjusted *P* values. Compared with the baseline percentage of participants at high risk of suicide, there was a statistically significant decrease in the percentage of those at high risk at the 3-month follow-up (*P*<.001). There was no statistically significant difference between the baseline and 5-week postbaseline values (*P*=.08) but there was a statistically significant reduction from the 5-weeks postbaseline value to the 3-month follow-up (*P*=.01).

#### Changes in Suicidal Ideation by Demographics

Significant reductions in the proportion of people experiencing suicidal ideation were observed at follow-up among female and gender-diverse participants, heterosexual and unsure or questioning participants, and those aged 16-18 years and 19-21 years (Multimedia Appendix 1 for full details).

### User Experience

Participants favorably rated their user experience of ReachOut, agreeing that it was relevant and accessible and that it helped them to understand their experiences and gave them practical help strategies and tools. See Table 1 for the percentages of endorsement for each user experience goal.

## Discussion

**Principal Findings**

This study aimed to explore the impact of ReachOut, an unstructured digital mental health intervention, on mental health outcomes and risk of suicide over time. The findings demonstrate that over a 3-month period, young people using ReachOut experienced a significant reduction in symptoms of depression, anxiety, and stress. Significant reductions in the proportion of participants at high risk of suicide were also observed.

The findings indicate that despite being a prevention and early intervention service designed for young people in mild-to-moderate levels of distress, the service is, in fact, also attracting young people in high levels of distress and with more complex presentations. A relatively large number of participants in this study had a history of mental health-related hospital admissions, which was substantially higher than the population estimates [45]. Further, 12.30% (194/1577) and 8.50% (134/1577) of the sample were at high risk of suicide at baseline and follow-up, respectively. ReachOut is not designed to be a crisis service or to meet the particular needs of young people at risk of suicide. However, we recognize that these young people are visiting ReachOut, perhaps unaware of our extent of service, and we acknowledge our duty of care to them. It is also probable that some visitors to ReachOut are aware of our extent of service but have had negative experiences with crisis support services. In these instances, there is an opportunity to leverage our position as a trusted support service to bridge the gap and build on their readiness to reengage with the support that is matched to their needs.

An important consideration for open-access websites and other eHealth tools is that while they can be targeted at particular levels of need, this does not necessarily mean that users will engage with them in the prescribed way. This gives rise to duty of care implications. As such, ReachOut is supported by a Clinical Advisory Group that supports the organization in implementing the duty of care and risk management framework. ReachOut is undertaking a program of continuous improvement, research, and partnerships with specialist support services to ensure young people with more complex presentations, particularly those at risk of suicide, are directed to the support that best matches their needs. A machine learning tool [46] has been developed to assess the risk within ReachOut’s peer support community. It triages and escalates posts that are deemed to be high-risk, whereupon staff moderators follow up and work closely with the young person and appropriate partner organizations (including emergency services) to keep them safe. Where deemed necessary, moderators edit or remove high-risk posts where there is a risk to the safety of the broader community. This triaging tool has been running successfully since 2016 and has been increasingly accurate in triaging posts in the community forum to support efforts to keep the community and moderators safe. A direct referral from ReachOut to Lifeline’s web chat service is currently being piloted and we are also undertaking a research project that explores the level of risk as a function of browsing behaviors, to support the automatic identification of young people who could benefit from more intensive support and targeting a persuasive intercept that encourages them to do so.

Although the improvements seen in mental health outcomes and suicide risk were relatively modest, they should not be discounted given the significant mental health needs of this cohort and the pronounced burden associated with mental illness during this developmental phase more broadly [47]. Reduction of symptoms of depression and anxiety of a similar magnitude has been reported in other published evaluations of mental health prevention programs [48-51], many of which are more resource-intensive, typically delivered face-to-face and often within school settings. Although the methodological differences of these studies prevent direct comparison of effects, the overall effects of ReachOut, a self-directed, brief, unstructured, and relatively inexpensive intervention, are promising alongside these more resource-intensive programs.

Many of the young people who participated in this study had previously sought help from a mental health professional, and a number of them had previous mental health hospital admissions. However, many did not rate their experience with mental health professionals as helpful. Digital interventions are uniquely positioned to provide alternative interim support to young people who may have had previous unfavorable experiences with traditional modes of care and to encourage them to reengage with more specialist support where appropriate. Although previous help-seeking experiences were poorly evaluated, participants rated their experience of ReachOut more favorably. These positive ratings can likely be attributed to the flexible and accessible mode of delivery, which mitigates a number of common barriers to help-seeking, and in part to the co-design methods employed in service and content development. ReachOut takes a participatory approach, co-designing services with diverse groups of current and/or prospective users, and adopts user-led experience goals to ensure that services are tailored for people with different needs. These findings, coupled with existing literature [35,52,53], present a strong argument for employing co-design methods in the development of youth mental health services. Furthermore, the positive ratings of ReachOut highlight the demand for web-based services among young people in high distress and underscore the importance of web-based services across the spectrum from early intervention to crisis services. Although more intensive, face-to-face treatment service modalities clearly have their place, it is important that early intervention and prevention are also prioritized to help reduce the incidence of common mental disorders early in the lifespan and maximize the efficiency of the limited resources available [54].

The participants in this study rated their user experience favorably in terms of both the relevance and accessibility of the program, and the *usefulness* of the intervention in providing them with a range of practical strategies and helping them understand their own experiences; however, the latter were not as positive as the former. As an open-access and completely self-directed service that services young people of varying ages, backgrounds, and levels of distress, it is likely that young people have varying levels of success in finding content that is relevant to their personal circumstances, given the vast array of issues and experiences covered in the site. ReachOut is currently exploring how personalization can optimize the experience of visitors to the site, helping them rapidly navigate to the most relevant information and support for them based on their needs and preferences, without having to wade through content that is not relevant or helpful. We hypothesize that a more personalized user experience may in turn result in higher ratings of usefulness in future evaluations of ReachOut.

Given the considerable health burden of mental health conditions in young people and the established barriers to accessing support [10,12,13], web-based mental health services offer a unique opportunity to address the needs of young people. Digital services are making rapid gains in the delivery of health and mental health care and are recognized for their acceptability, scalability, and reach [55]. Digital services can overcome common barriers faced by young people when accessing mental health services, including stigma and preference for self-reliance [10], provide support to large numbers of young people in a highly accessible and youth-friendly manner, and offer a unique opportunity to access hard-to-reach groups. Compared with traditional services, digital services, provided they are effective and accessed by large numbers of people, are cost-effective and may provide relief from symptoms in their own right, while also facilitating onward referral when required [55]. We found similar improvements in mental health symptoms across various demographic groups, including by age, gender, and sexual orientation. We hypothesize that the lack of notable differences between these groups is related to the involvement of diverse young people in the development of service resources and the flexibility and self-directed nature of the intervention. Young people from all backgrounds are involved in the co-creation of information, support, and tools for ReachOut. In addition, the unstructured nature of ReachOut allows young people to navigate and select information and/or support that may be relevant to them from a wide variety of topics and diverse perspectives. The result is a flexible intervention that is adaptable to the individual, and yet can be scaled up to reach large numbers of young people in need.

### Limitations and Future Directions

Overall, the study provided valuable insights into the potential of an unstructured digital intervention; however, this study is not without its limitations. First, participants self-selected into the study, and there were minimal eligibility criteria other than age, location, and current use of ReachOut. Although all participants were ReachOut users, there was no minimum requirement of usage for the duration of the study. Therefore, it is difficult to specifically attribute dosage patterns to improvement in mental health and suicide risk. Furthermore, one goal of the intervention was to facilitate access to professional help, and as such, the use of professional mental health services alongside ReachOut may have resulted in greater reductions observed in mental health scores over time. The possible impact of other help-seeking avenues reduces our confidence in the attribution of causality to the intervention. In addition, this sample had complex mental health needs that may not be representative of young people across the mental health spectrum but rather of those in high distress. Self-report measures were used to capture mental health status and no observer-rated assessments were used. The majority of participants in the study were female, thus limiting generalizability; however, there is also a skew toward young female users within the wider ReachOut user population and the profile of young people who seek help for psychological distress in Australia [13].

Furthermore, while the results indicate significant improvements in mental health and suicide risk among young people, the lack of a control group precludes the definitive attribution of these outcomes to their use of ReachOut. Although RCTs represent the gold standard in evaluating some treatment outcomes, there can be challenges in implementing these studies within the context of a brief, unstructured, and self-directed digital intervention that operates within a dynamic service environment and is rapidly responding to the changing needs of young people. Further, the pace at which digital technology is evolving requires a faster delivery of research and evaluation insights to ensure that these are timely and relevant; the significant time investment of RCT designs is often not conducive to the fast-paced innovation cycle. More rapid sources of evidence are therefore required in addition to RCTs to support interventions to adapt to new technologies and evolving user needs. Further, as others have noted, a favorable outcome in an RCT does not always imply that the intervention will have a meaningful impact in the real world, especially in the digital space [38]. There have been a number of web-based and app-based interventions that have demonstrated favorable results through RCTs [56-58]; however, there has been little consideration of the application of these interventions in the natural environment, in which usage patterns and dosages are unknown, and the environments in which these are implemented are uncontrolled [38,59]. Despite these challenges, discrete components of the ReachOut service offering have been subjected to controlled trials [41]. Future evaluation research that includes a control group would be valuable for drawing more definitive conclusions regarding the attribution of any observed changes to the intervention.

It is also important to consider that *spontaneous* remission (ie, remission of symptoms that are not related to a given treatment) may account for a proportion of the improvements in mental health and suicide risk observed over the 3-month study period. Without a control group, it is difficult to estimate the influence of spontaneous remission on these findings. There is currently limited evidence regarding the rates of spontaneous remission in young people. However, a meta-analysis that examined *remission* in untreated depression, defined as ‘clinically significant improvement’, found that 23% of adults remitted at 3 months, with a higher remission rate (not mentioned in the paper) observed in children and adolescents [60]. On the basis of this, we might conservatively estimate that between 20% and 30% of adolescents with an affective disorder spontaneously remit with the passage of time, and it is therefore possible that a proportion of the cases that experienced improvements in mental health outcomes would have experienced it anyway, even in the absence of the support they accessed from ReachOut. However, the level of reduction in symptoms that was observed, coupled with the young people’s positive endorsement of user experience goals as well as a self-report of the subjective impact (ie, how ReachOut helped them) in the qualitative data (see [61]), suggests that ReachOut did contribute to the improvements seen in mental health and suicide risk, although different research designs are required to establish more definitively the extent of this contribution. As noted above, a study design that involves a matched control group would allow for a more thorough investigation of the potential impact of spontaneous remission on the observed effects. This would also allow for an exploration of whether there were any possible study effects associated with completing the evaluation surveys, as opposed to engaging with the intervention per se.

A final limitation of the study is the lack of objective data on the young people’s activity on ReachOut, which precludes the examination of engagement variables as a potential mediator of impact. Future research on ReachOut will build on this study by capturing the users’ browsing data to explore the impact of different components of the intervention on mental health outcomes and the dosage effects. It is hoped that this research will further elucidate the critical factors of unstructured interventions that lead to improvements, including any influences of dose (frequency and duration of use), modality of delivery, and content format.

Despite these possible limitations, this study provided preliminary evidence of promise for a brief, unstructured, self-directed digital intervention to support young people’s mental health and well-being. The large potential for digital interventions for youth mental health and well-being is widely acknowledged [62], and given the substantial evidence of the acceptability, effectiveness, and cost-effectiveness of digital interventions, there is a clear rationale to invest in the development and evaluation of evidence-based digital services for youth mental health.

### Conclusions

Digital mental health interventions can address barriers to access and provide services to young people in need at a population level, and there is growing evidence that they are effective in improving mental health outcomes among youth. Specifically, these findings demonstrate the promise of an unstructured digital mental health intervention, ReachOut, in reducing depression, anxiety, and stress symptoms, and risk of suicide in young people. Although ReachOut is primarily intended for prevention and early intervention, the findings from this study highlighted that the service is attracting young people in high levels of distress. Despite the considerable mental health needs of this cohort, significant improvements in all outcomes were observed and young people rated their experience of using ReachOut highly. Our findings, coupled with existing research, indicate that unstructured digital mental health services are not only accessible and acceptable but also have the potential to cater to the diverse mental health needs of young people, in a way that other services are unlikely to be able to do because of limited resources and ability to scale. Future research that involves a comparison group and the collection of user data to explore dose-response effects is needed to confirm and expand on these findings.

## Appendix

Multimedia Appendix 1 Changes in risk of suicide over time split by gender, sexual orientation, and age group.

## Abbreviations

ANOVA: analysis of variance
DASS-21: Depression, Anxiety and Stress Scale-21
RCT: randomized control trial
SIQ: Suicidal ideation Questionnaire
