# Supplementary material for: Evaluation of ReachOut.com, an Unstructured Digital Youth Mental Health Intervention: Prospective Cohort Study
Source: JMIR Ment Health. 2020 Oct 15;7(10):e21280. doi: 10.2196/21280 (PMC7596653; doi:10.2196/21280)
Supplement: Multimedia Appendix 1 [file mental_v7i10e21280_app1.docx]

**Appendix 1**

**Changes in risk of suicide over time split by gender, sexual orientation, and age group.**

The file was split by group and analyses were run as per main analyses to look at changes in proportion of young people at risk of suicide by gender, sexual orientation, and age group across time.  A summary of the results follow:

**Gender:**

- Males - no significant reductions in suicide risk across time (T1=9.7%; T3=12.3%; T4=8.0%), χ^2^(2)=3.38, *P*=.185
- Females - significant reduction in suicide risk from baseline to five week post-baseline, and from baseline to three month follow-up (T1=12.4%; T3=9.9%; T4=8.7%), χ^2^(2)=20.48, *P*<.001
- Other gender identities - significant reduction in suicide risk from five weeks post-baseline to the three month follow-up (T1=15.8%; T3=17.1%; T4=6.6%), χ^2^(2)=8.14, *P*=.017

**Sexual orientation:**

- Heterosexuals - significant reduction in suicide risk from baseline to five week post-baseline, and from baseline to three month follow-up (T1=8.5%; T3=8.3% T4=6.1%), χ^2^(2)=10.26, *P*=.006
- Unsure and questioning - significant reduction in suicide risk from baseline to five week post-baseline, and from baseline to three month follow-up (T1=26.3%; T3=17.5%; T4=13.9%), χ^2^(2)=12.38, *P*=.002
- Gay and lesbian (T1=15.7%; T3=16.9%; T4=15.7%; χ^2^(2)=0.10, *P*=.958), bisexuals (T1=17.2%; T3=12.5%; T4=13.0%; χ^2^(2)=3.56, *P*=.169), and other sexual identities (T1=17.5%; T3=14.3%; T4=9.5%; χ^2^(2)=5.85, *P*=.054) showed no significant reduction in suicide risk across time.

**Age-groups:**

- 16-18 years - significant reduction in suicide risk from baseline to three month follow-up (T1=14.3%; T3=12.3%; T4=9.6%), χ^2^(2)=14.57, *P*=.001
- 19-21 years - significant reduction in suicide risk from baseline to five week post-baseline, and from baseline to three month follow-up (T1=11.9%; T3=7.9%; T4=7.2%), χ^2^(2)=11.83, *P*=.003
- 22-25 years - no significant reduction in suicide risk across time (T1=8.9%; T3=10.2%; T4=7.6%), χ^2^(2)=3.19, *P*=.203
